# Vaccine hesitancy for coronavirus SARS-CoV-2 in Varanasi India

**DOI:** 10.3389/fpubh.2022.892584

**Published:** 2022-10-05

**Authors:** Utkarsh Srivastava, Avanish Kumar Tripathi, Jagjeet Kaur, Sabita Devi, Shipra Verma, Vanya Singh, Debashruti Das, Prajjval Pratap Singh, Rahul Kumar Mishra, Nikhil A. Kumar, Vijaya Nath Mishra, Pradeep Kumar, Vandana Rai, Rakesh Tamang, Prashanth Suravajhala, Rakesh Pandey, Gyaneshwer Chaubey

**Affiliations:** ^1^Anthropology Division, Department of Sociology, Faculty of Social Sciences, Banaras Hindu University, Varanasi, India; ^2^Cytogenetics Laboratory, Department of Zoology, Banaras Hindu University, Varanasi, India; ^3^Department of Neurology, Institute of Medical Science, Banaras Hindu University, Varanasi, India; ^4^Department of Biotechnology, VBS Purvanchal University, Jaunpur, India; ^5^Department of Zoology, University of Calcutta, Kolkata, India; ^6^Amrita School of Biotechnology, Amrita Vishwa Vidyapeetham, Amritapuri, Kerala, India; ^7^Department of Psychology, Faculty of Social Sciences, Banaras Hindu University, Varanasi, India

**Keywords:** vaccine hesitancy, SARS-CoV-2, coronavirus, North India, Varanasi

## Abstract

With the rollout of the world's largest vaccine drive for SARS-CoV-2 by the Government of India on January 16 2021, India had targeted to vaccinate its entire population by the end of 2021. Struggling with vaccine procurement and production earlier, India overcome these hurdles, but the Indian population still did not seem to be mobilizing swiftly toward vaccination centers. The severe second wave has slowed the vaccination pace and was also one of the major contributing factors to vaccine hesitancy. To understand the nature of vaccine hesitancy and its underlying factors, we conducted extensive online and offline surveys in Varanasi and adjoining regions using structured questions. Most respondents were students (0.633). However, respondents from other occupations, such as government officials (0.10), have also participated in the study. Interestingly, most people (0.75) relied on fake news and did not take COVID-19 seriously. Most importantly, we noticed that a substantial proportion of respondents (relative frequency 0.151; mean age 24.8 years) reported that they were still not interested in vaccination. We observed a significant association between vaccine hesitancy and socioeconomic status (χ^2^ = 307.6, *p* < 0.001). However, we failed to detect any association between vaccine hesitancy and gender (χ^2^ = 0.007, *p* > 0.5). People who have neither been vaccinated nor have ever been infected may become the medium for spreading the virus and creating new variants, which may lead to the vaccine-resistant variant. We expect this extensive survey to help the Government upgrade their vaccination policies for COVID-19 in North India.

## Introduction

COVID-19 has impacted our lives in multiple ways (1, 2). Studies have observed age and comorbidity as strongly associated factors for the disease severity (3–6). Moreover, the long-COVID and post-COVID complications have added another complexity to this disease (7–11). Since this disease is new, information related to it is not very concrete. With the latest research accumulating daily (3), the WHO and government guidelines have changed substantially. These changes have mystified the general population (3, 12, 13). Thus, several local rumors against the vaccination drive have surfaced in the population (14, 15). Since the flow of information in Indian society heavily depends upon oral transmission, i.e., word-of-mouth, many people are afraid to visit vaccination centers (16).

India began the vaccination drive on January 16 2021. Only ~200,000 cases were active during this time, and most Indians had overcome the trauma of the first wave (17). With repeated encouragement from the Government, India has achieved 22 million doses per day by the end of March 2021 (18). This number increased exponentially during the first week of April 2021, when the Government decided to vaccinate everyone above 45 years of age (19). However, this was also the time of the beginning of the second wave (20, 21). Due to the severe second wave, the daily vaccine doses administered, which were more than 35 million a day till April 13 2021, have been reduced to < 15 million a day just after a month (18).

Moreover, leaders from several political parties have released public statements against vaccination (22). Those mentioned above appear to significantly contribute to the reduced vaccination rate after the second wave (10). Recent studies on vaccine hesitancy have highlighted the significant reasons and rigorous vaccination campaigns to overcome the problem (14, 23–29). The concern about the side effects was highlighted, and it has been shown that at the global level, females are more hesitant than males (28). Indian society is segregated into various castes and tribal populations. Our recent study has reported that the susceptibility of several smaller tribal populations is significantly higher than the other populations (30). A study on social affiliation and vaccine hesitancy has suggested 3.5 times higher vaccine hesitancy among Scheduled caste populations (31). Thus, it is pertinent that low education and lower socioeconomic status is the primary cause of vaccine hesitancy (23, 26, 28, 29).

So far, the Varanasi and adjoining regions have not been surveyed for vaccine hesitancy. Therefore, to understand vaccine hesitancy in North India, we have systematically uncovered the cause. Some empirical evidence is much needed to understand the nature and cause of the vaccine hesitancy to suggest a potential psychosocial intervention to help the North Indian policymakers and immunization staff to overcome this key hurdle in immunization against COVID-19. To understand the nature and causes of vaccine hesitancy among North Indians, we conducted an extensive survey in Varanasi and adjoining regions. We followed a questionnaire-based survey approach to uncover the factors that inculcate vaccine hesitancy (Table 1). We presented structured questions with a predefined set of responses for each question.

**Table 1:** Respondents frequency (with 95% CI) on multiple choice questions investigated during the survey; *n* = number of samples.

		**Total Freq. (95%CI)**	**Male Freq. (95%CI)**	**Female Freq. (95%CI)**

1 What is Coranavirus?		n=727	n=425	n=302
	Natural pandemic	0.317 (0.287-0.349)	0.335 (0.295-0.379)	0.293 (0.249-0.342)
	Lab made virus	0.083 (0.066-0.103)	0.085 (0.064-0.114)	0.079 (0.056-0.111)
	Biological weapon	0.123 (0.102-0.146)	0.081 (0.06-0.109)	0.177 (0.141-0.219)
	Global conspiracy	0.433 (0.4-0.466)	0.465 (0.420-0.509)	0.391 (0.343-0.442)
	Government weapon	0.039 (0.028-0.054)	0.033 (0.021-0.053)	0.060 (0.040-0.089)
2 What does the corona vaccine do?		n=533	n=317	n=216
	Makes you impotent	0.006 (0.002-0.016)	0.003 (0.001-0.017)	0.009 (0.003-0.033)
	Prevents corona	0.899 (0.87-0.921)	0.905 (0.868-0.933)	0.889 (0.84-0.924)
	Population control	0.019 (0.01-0.034)	0.019 (0.009-0.041)	0.019 (0.008-0.047)
	Makes you emotionless	0.019 (0.01-0.034)	0.019 (0.009-0.041)	0.019 (0.008-0.047)
	Leads to death	0.058 (0.041-0.081)	0.054 (0.034-0.084)	0.065 (0.039-0.106)
3 What was the role of the government during the corona pandemic?		n=813	n=482	n=331
	Can be improved	0.389 (0.356-0.423)	0.351 (0.309-0.394)	0.444 (0.392-0.498)
	Irresponsible attitude	0.219 (0.192-0.249)	0.241 (0.205-0.281)	0.187 (0.149-0.233)
	Satisfactory	0.097 (0.0179-0.119)	0.087 (0.065-0.116)	0.112 (0.082-0.15)
	Very good	0.111 (0.091-0.134)	0.116 (0.091-0.148)	0.103 (0.075-0.14)
	Worrying	0.185 (0.159-0.213)	0.205 (0.172-0.244)	0.154 (0.119-0.197)
4 What was the public's role in spreading coronavirus (SARS-CoV-2) related informations?		n=848	n=480	n=368
	Relied on rumors	0.317 (0.287-0.349)	0.335 (0.295-0.379)	0.293 (0.249-0.342)
	Agreed with government	0.083 (0.066-0.103)	0.085 (0.064-0.114)	0.079 (0.056-0.111)
	Followed health instructions	0.123 (0.102-0.146)	0.081 (0.06-0.109)	0.177 (0.141-0.219)
	Didn't take seriously	0.433 (0.4-0.466)	0.465 (0.420-0.509)	0.391 (0.343-0.442)
	Took seriously	0.039 (0.028-0.054)	0.033 (0.021-0.053)	0.060 (0.040-0.089)
5 Which of the following steps would help stop the infection of coronavirus?		n=1218	n=721	n=497
	Total lockdown	0.250 (0.226-0.275)	0.247 (0.217-0.280)	0.256 (0.219-0.296)
	Partial lockdown	0.089 (0.075-0.107)	0.097 (0.078-0.121)	0.078 (0.058-0.106)
	Personal consciousness and awareness	0.380 (0.353-0.407)	0.368 (0.333-0.403)	0.400 (0.358-0.444)
	Total vaccination	0.278 (0.254-0.304)	0.288 (0.257-0.323)	0.266 (0.229-0.306)
6 How do you view the health management of India during COVID-19 second wave?		n=656	n=370	n=286
	Good	0.064 (0.048-0.085)	0.065 (0.044-0.095)	0.063 (0.040-0.097)
	Very Good	0.046 (0.032-0.065)	0.041 (0.025-0.066)	0.052 (0.032-.085)
	Satisfactory	0.168 (0.141-0.198)	0.157 (0.123-0.197)	0.182 (0.141-0.231)
	Unsatisfactory	0.410 (0.373-0.448)	0.422 (0.372-0.473)	0.395 (0.340-0.453)
	Average	0.313 (0.278-0.349)	0.316 (0.271-0.365)	0.308 (0.257-0.364)
7 Would you prefer to get vaccinated?		n=603	n=337	n=266
	Yes	0.849 (0.818-0.875)	0.849 (0.806-0.883)	0.850 (0.802-0.887)
	No	0.151 (0.125-0.182)	0.151 (0.117-0.194)	0.150 (0.113-0.198)
8 Did you take the COVID-19 test?		n=603	n=337	n=266
	Yes	0.388 (0.350-0.428)	0.418 (0.367-0.472)	0.350 (0.295-0.409)
	No	0.612 (0.572-0.650)	0.582 (0.528-0.633)	0.650 (0.591-0.705 )
9 What was the test result?		n=317	n=175	n=142
	Positive	0.388 (0.350-0.428)	0.418 (0.367-0.472)	0.350 (0.295-0.409)
	Negative	0.612 (0.572-0.650)	0.582 (0.528-0.633)	0.650 (0.591-0.705 )
10 Which vaccine are you aware of?		n=1274	n=759	n=515
	All	0.038 (0.029-0.050)	0.025 (0.016-0.039)	0.056 (0.40-0.080)
	Covishield	0.349 (0.323-0.375)	0.358 (0.325-0.393)	0.334 (0.295-0.376)
	Covaxin	0.376 (0.350-0.403)	0.364 (0.330-0.398)	0.394 (0.353-0.437)
	Sputnik-V	0.238 (0.215-0.262)	0.253 (0.223-0.285)	0.216 (0.182-0.253)
11 Will vaccination prevent COVID-19 lifelong?		n=632	n=278	n=351
	Yes	0.082 (0.063-0.106)	0.119 (0.086-0.162)	0.054 (0.035-0.083)
	No	0.441 (0.403-0.480)	0.572 (0.513-0.629)	0.342 (0.294-0.393)
	Not Sure	0.472 (0.433-0.511)	0.572 (0.513-0.629)	0.396 (0.346-0.448)

## Methodology

### Participants

The study was conducted on a relatively sizeable incidental sample of participants (N =603 Males = 337, Females = 266) in the age range of 18 to 40 years (mean age =26.9; SD = 4.4). Only those respondents were included in the study who volunteered themselves and consented to participate in the study. We have also conducted an offline survey together with the online survey (telephonic interview). In the analysis procedures, we have anonymized the participants. The Ethical committees of Banaras Hindu University, Varanasi and VBS Purvanchal University, Jaunpur, India, have approved the study. Though the attempt was made to recruit participants from different occupational backgrounds, most respondents were students (0.633) with relatively few government employees (0.10).

### Materials and procedure

We conducted a questionnaire-based survey (Table 1) consisting of 11 questions related to awareness about the COVID-19 pandemic, its spread and vaccination. The survey was primarily conducted through an online platform. The telephonic survey was also done to reach people from rural areas (who could not use the online platform). This was done to understand their attitudes and perspectives regarding the COVID-19 scenario (which is equally crucial for urban people). Such a telephonic survey was done on rural people and frontline health workers to learn about the vaccination drive and related hesitancy among rural masses.

We divided our survey into two sections: Population demographic information- age, gender, and occupation. The 11 questions deal with vaccine hesitancy-related issues (Table 1). Multiple options were supplied in an objective direction. In the second section, participants of telephonic interviews were the frontline health workers, including CHO (Community Health Workers), ANM (Auxiliary Nurse Midwives), ASHA (Accredited Social Health Activists) and ASHA *Sangini*. They have maintained their record and have shared with us their observations. The interview was structured, and the main emphasis was on the two questions that were asked-

Q.1:- What is the primary restraint among people of rural India to participate in COVID-19 vaccination?

Q.2:- How do you see the vaccination drive in your area, and if you have to suggest a few reasons, kindly list them to make the vaccination drive more inclusive and widespread. We tend to use it as additional data to have a better and broadened look over the conclusion drawn from our study and whether it complies with it.

Apart from the health workers, we also did a second telephonic interview with people from rural areas. This interview was also structured, and it consisted of two questions-

Q.1:- Do you want to get vaccinated?

Q.2:- If not, then why?

### Statistical analyses

The frequency of each response was calculated with a 95% CI (Table 1). The barplot with the 95% CI was drawn separately for the male and female participants. The per month earnings of each participant were recorded in the four categories [<5000 (1); 5001–10000 (2); 10001–50000 (3); 50001–100000 (4])]. The gender of the respondent was recoded to 0 (male) and 1 (female), and vaccine hesitancy answers were recoded to 0 and 1 (No and Yes). The chi-square (χ^2^) and logistic regression statistical analyses were conducted using SPSS (ver.26). For statistical significance, a two-tailed *p*-value test was performed.

## Results

In order to understand the vaccination drive in a region, it is necessary to focus on the local hurdles behind vaccine hesitancy. Our questionnaire was designed to reflect the mass feeling about the nature of the virus, the second wave, comments on measures taken by the Government during the second wave, and rumors leading to vaccine hesitancy (Table 1). Apart from the highly infective virus variants during the second wave (32), the role of the public was also concerning. A large proportion of participants (0.75) either relied on rumors or did not take the virus seriously. Moreover, according to the respondents, when asked what steps would help prevent the coronavirus, most people think that total vaccination and personal consciousness (0.658) will be a better tool than lockdowns (Table 1).

An exciting result that our study yielded is that in some questions, there was a significant difference (two-tailed *p*-value < 0.001) in the responses between male and female respondents (Supplementary Figure 1). For example, a significantly lower number of females think that the coronavirus is a lab-made or biological weapon. In contrast, more females think COVID-19 is a natural pandemic (two-tailed *p*-value < 0.001) (Table 1). Similarly, more females followed the health instructions than the males (two-tailed *p*-value < 0.001).

During the survey, many participants had an impression that the vaccine relates to introducing the second wave. Therefore, we first investigated the vaccine hesitation during the second wave (Table 1 and Supplementary Figure 1). We have looked at the vaccination data during March-June 2021 (18). We found a major vaccination dip during the second wave (two-tailed *p* < 0.0001).

**Figure 1:**
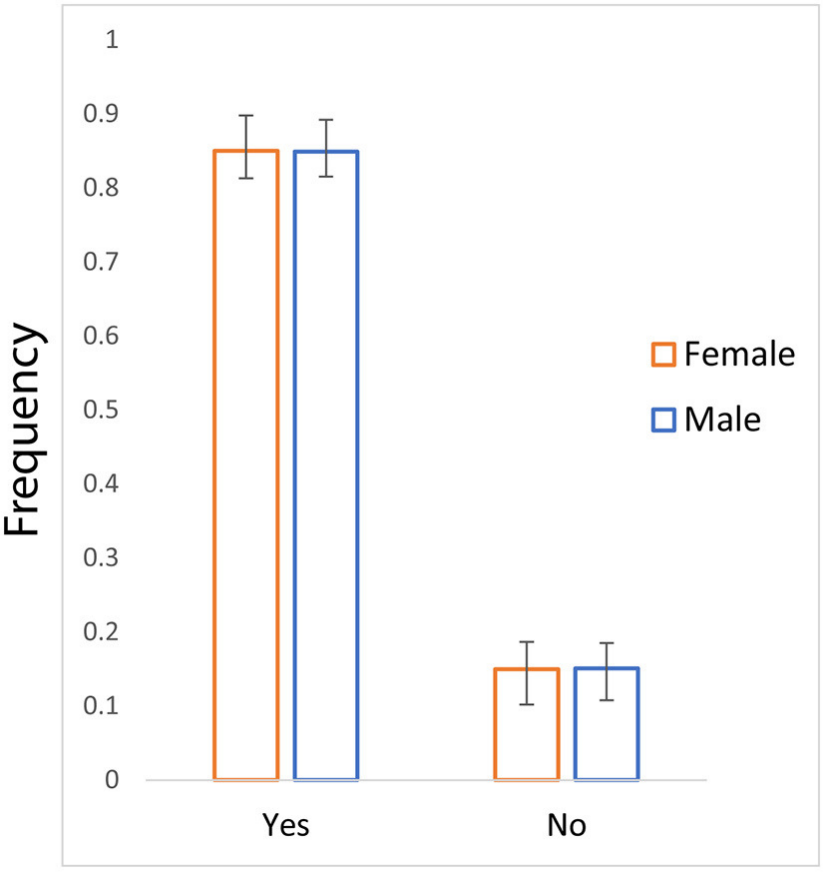
The bar plot of frequency (with 95%CI) showing vaccine hesitancy for male and female participants.

From our research, we found that a large proportion of people (0.849) prefer to get vaccinated. They are aware of vaccines (0.962) and know that the vaccine for SARS-CoV-2 prevents COVID-19 (0.899). However, a substantial proportion of people reported that they would refrain from vaccinating (0.15) (Figure 1). Remarkably, the vaccine hesitancy ratio was similar for both male and female participants. The vaccine acceptance among the studied cohort in India is significantly (two-tailed *p* < 0.0001) higher than the global data (28). It is worthwhile to mention here that the trend of vaccine willingness in the Indian community is similar to the data of Bangladesh (29, 33); nevertheless, the educated community in India is significantly (two-tailed *p* < 0.0001) well aware and is at a greater acceptance.

Our statistical tests have yielded a highly significant association between vaccine hesitancy and the economic status of the participants (χ^2^ = 307.6, *p* = 0.000). Nevertheless, the gender-biased association of vaccine hesitancy has not been observed in our survey (χ^2^ = 0.007, *p* = 0.933) (Table 2). The logistic regression analysis supported the strong association of vaccine hesitancy with the economic status of the respondents (Table 3).

**Table 2:** Vaccine hesitancy according to the demographics.

**Demographics**	**Vaccine Hesitancy**		
	**Pearson Chi Square (**χ^2^**)**	**Asymptotic Sig (2-sided)**	**Cramer's V**
Income	307.6	0.000	0.714
Gender	0.007	0.933	0.003

**Table 3:** Determinants of vaccine hesitancy.

**Determinants**	**Vaccine Hesitancy**		
	**Odds Ratio (95%CI)**	**Wald**	***p*-value**
Income	61.851 (27.351–139.867)	98.163	0.000
Gender	1.028 (0.544–1.942)	0.007	0.933

## Discussion

The present study effectively contributes to the vaccine hesitancy in district Varanasi and adjoining regions of India. We focused primarily on the educated people with a list of a questionnaire. Our findings show a significant (two-tailed *p* < 0.0001) hesitancy among males and females (Figure 1). Strikingly, the Indian cohort studies here had lower vaccine hesitancy than the global data, likely due to our cohort structure. Our cohort in the present study was overwhelmed by people with higher education.

India's vaccine drive fluctuated with a significant drop during the second wave significantly (two-tailed *p*-value < 0.0001). The most crucial reason for this fall was vaccine hesitancy rumors. Our interview and observation found that people ran for the vaccine as soon as the second wave started to spread. It resulted in enormous rush to the vaccination centers. Many have been infected due to large gatherings at the vaccination centers. This has created confusion in society that the people are being infected after taking vaccines (Table 1 and Supplementary Figure 1). Thus, vaccines are not helping to stop the infection. The spread of this rumor through word-of-mouth has reduced the vaccinations significantly (two-tailed *p-*value < 0.0001) (18). During the second wave, the daily immunization was low when the positive test rate was at its peak. However, it must be understood that it takes 3–4 weeks to develop the effective antibodies after the vaccination (34).

So far, in the SARS-CoV-2 evolution, we have seen that this virus can create more hazardous variants with time (32). Moreover, we are fortunate that no variant has been found that completely evades vaccine-induced immunity. Still, with a large number of vaccination, a non-vaccinated pool may provide a reservoir for the virus to multiply and mutate. Thus, it may offer the opportunity to emerge new variants. Moreover, the selection pressure on the virus against the background of a primarily vaccinated population may favor a variant that will be resistant to the vaccine. Therefore, the real danger is from those who have never been vaccinated or infected before. Such people will provide ground for a new variant of the virus. If it develops immunity to the vaccine, it will be a major setback in controlling the epidemic. The progress we have made against this pandemic will be lost.

Consistent with the previous observations, our multiple statistical analyses confirmed the strong correlation of vaccine hesitancy with the economic status of the participants (Tables 2, 3). Whilst, the gender-specific difference has not been observed, which is likely due to the nature of our cohort, where most of the respondents are well educated.

## Limitations and future perspectives

We caution that the cohort used in our study is overwhelmed by educated people. Therefore, the hesitancy frequency observed in this study may capture the lower bound data of vaccine hesitancy in North India. Further, we add that a retrospective study following face-to-face or structured telephonic interviews with a qualitative approach such as thematic analysis may bring further insight into the dynamics of vaccine hesitancy among Indians. Similarly, post-second wave vaccine hesitancy status also needs to be explored using the same interview format and contrasted with the retrospective data to understand the extent of vaccine hesitancy and changing factors. Since we have used a structured questionnaire with a predefined response format, the study is fraught with the danger of the researchers' subjective biases as the researchers' proposed factors for vaccine hesitancy were limited. The open-ended questions for listing the reasons for vaccine hesitancy may bring newer insights and additional aspects of vaccine hesitancy that could not be foreseen by us while framing the response to the question of vaccine hesitancy.

## Conclusions

In conclusion, our study adds systematic knowledge on various potential factors related to the COVID-19 vaccine hesitancy among North Indians. During the second wave, most people in North India relied on fake news. > 65% population opposed total lockdown. A significant number of females were better at following the official health instructions. Vaccine hesitancy is found among 15% of the studied cohort. Consistent with the previous studies, we have also observed a significant correlation between vaccine hesitancy and socioeconomic status. In contrast, we did not find any correlation between vaccine hesitancy and gender. Thus, a region-specific policy is needed for COVID-19 vaccination in North India.

## Data availability statement

The datasets presented in this study is provided in Table 1 and Supplementary File.

## Ethics statement

The studies involving human participants were reviewed and approved by VBS Purvanchal University Jaunpur and Banaras Hindu University, Varanasi, India. The patients/participants provided their written informed consent to participate in this study.

## Author contributions

GC conceived and designed this study. US, AT, JK, SD, SV, VS, DD, PPS, RM, NK, VM, PK, VR, RT, PS, and RP collected the data and also conducted the online and offline surveys. US, AT, PPS, GC, DD, PK, RT, PS, and VR analyzed the data. GC, US, AT, JK, SD, SV, RT, PS, and RP wrote the manuscript from the inputs of other co-authors. All authors contributed to the article and approved the submitted version.

## Funding

This work was supported by the ICMR ad-hoc grant (2021-6389). GC and VM are supported by Faculty IOE grant BHU (6031). PPS was supported by CSIR fellowship, Govt. of India.

## Conflict of interest

The authors declare that the research was conducted in the absence of any commercial or financial relationships that could be construed as a potential conflict of interest.

## Publisher's note

All claims expressed in this article are solely those of the authors and do not necessarily represent those of their affiliated organizations, or those of the publisher, the editors and the reviewers. Any product that may be evaluated in this article, or claim that may be made by its manufacturer, is not guaranteed or endorsed by the publisher.
