# Supplementary figures and images for: Vaccine hesitancy for coronavirus SARS-CoV-2 in Varanasi India
Source: Front Public Health. 2022 Oct 5;10:892584. doi: 10.3389/fpubh.2022.892584 (PMC9581394; doi:10.3389/fpubh.2022.892584)

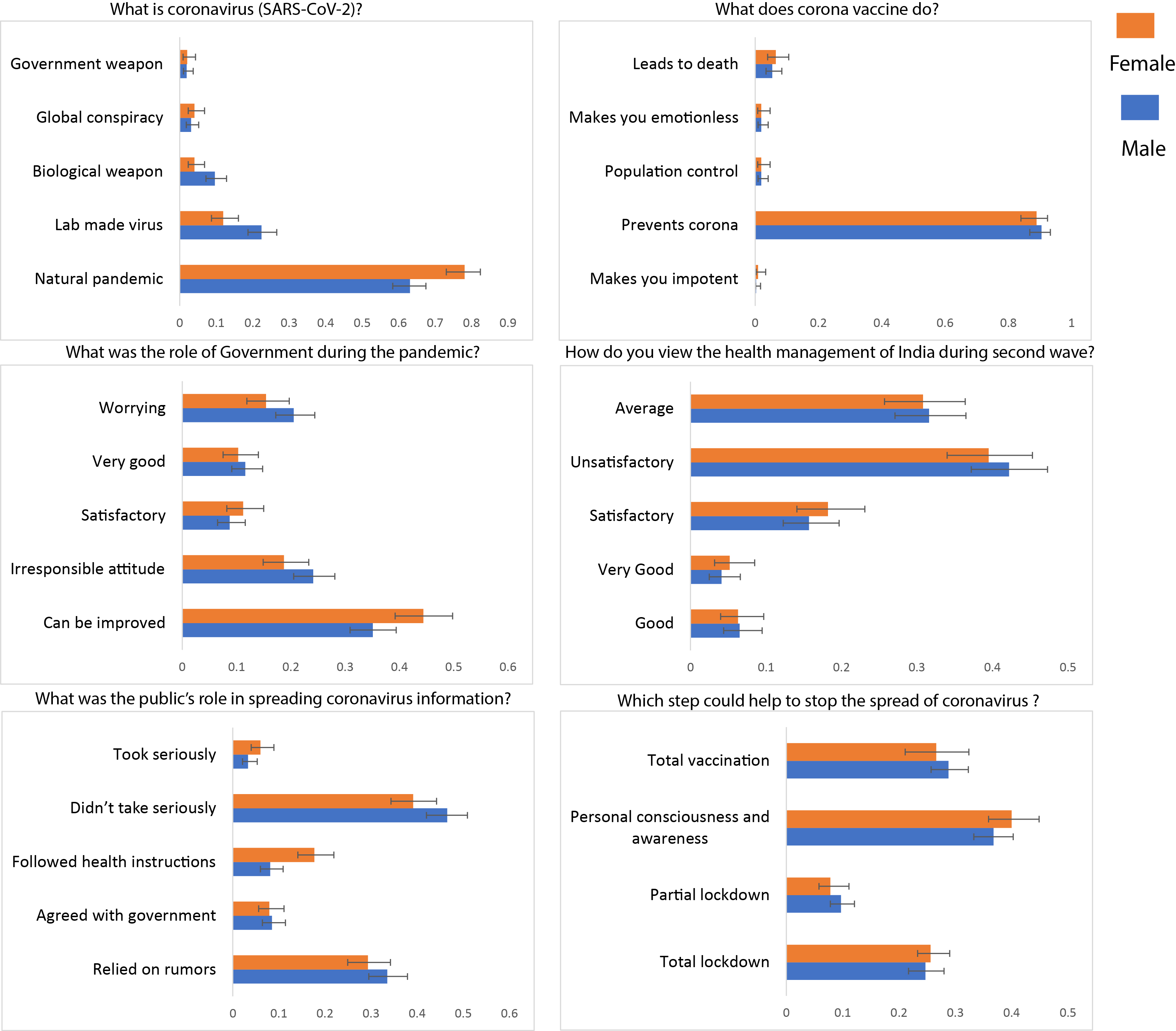

Supplement: Supplementary file 1 [file Image_1.JPEG]
